# Full-Length Recombinant Human SCF^1-165^ Is More Thermostable than the Truncated SCF^1-141^ Form

**DOI:** 10.1371/journal.pone.0103251

**Published:** 2014-07-25

**Authors:** Yui-Ping Weng, Wen-Yen Ku, Ming-Han Wu, Ya-li Tsai, Chi-Yu Chen, Chun-An Kuo, Lynn L. H. Huang

**Affiliations:** 1 Graduate Institute of Biological Science and Technology, Chung Hwa University of Medical Technology, Tainan, Taiwan; 2 Department of Biological Science and Technology, Chung Hwa University of Medical Technology, Tainan, Taiwan; 3 Institute of Biotechnology, Cheng Kung University, Tainan, Taiwan; University of San Francisco, United States of America

## Abstract

Human stem cell factor initiates a diverse array of cellular responses, including hematopoiesis, cell proliferation, differentiation, migration and survival. To explore the relationship between its structure and function, we produced recombinant soluble human stem cell factor^1–165^ (wild type) and human stem cell factor^1–141^ (C-terminal truncated) in a yeast expression system and compared their biological activities and thermal stabilities. The biological activity of the two proteins was measured as a function of TF-1 cell viability and effects on downstream signaling targets after incubation. We found that these proteins enhanced cell viability and downstream signaling to a similar extent, in a dose-dependent manner. The biological activity of recombinant human stem cell factor^1–165^ was significantly greater than that of recombinant human stem cell factor^1–141^ after heating the proteins (100 ng/mL) at 25–110°C for 10 minutes (P<0.05 for all temperatures). In addition, circular dichroism spectral analysis indicated that β-sheet structures were altered in recombinant human stem cell factor^1–141^ but not recombinant human stem cell factor^1–165^ after heating at 90°C for 15 or 30 min. Molecular modeling and limited proteolytic digestion were also used to compare the thermo stability between human stem cell factor^1–165^ and human stem cell factor^1–141^. Together, these data indicate that stem cell factor^1–165^ is more thermostable than stem cell factor^1–141^.

## Introduction

Human stem cell factor (hSCF) is a glycoprotein cytokine that induces Kit activity. The effects of SCF are exerted through at least 4 intracellular pathways and involve Src family members, phosphatidylinositol-3-kinase, the Janus family of protein tyrosine kinases (Jak), and the Ras-Raf-mitogen activated protein (MAP) kinase cascade. These pathways mediate a number of cellular processes, including gene transcription, proliferation, differentiation, survival, metabolic homeostasis, melanin pigmentation, development, and cell migration [1]–[10].

SCF is expressed as two different isoforms composed of 220 and 248 amino acids. These membrane-associated proteins are generated via alternative splicing of the same RNA transcript [11], the latter of which includes a proteolytic cleavage site in exon six. Cleavage at this site releases the extracellular portion of the protein from the membrane [12], [13], becoming the soluble form of SCF. Both the membrane-associated and soluble (SCF^1–165^) forms are biologically active [14]. The core of the protein required for activity comprises residues 1–141 and is reported to bind and activate the receptor Kit [15]–[17]. The function of the C-terminal domain of SCF^1–165^ is not yet known. Soluble SCF functions as a non-covalently associated homodimer, but the majority of SCF exists as a monomer under physiological conditions [18]. Each SCF monomer contains two intra-chain disulfide bridges (Cys4–Cys89 and Cys43–Cys138) that are required for its activity [16]. SCF monomers can be glycosylated at residues Asn65, Asn72, and Asn120 [13], [19].

SCF is involved in a wide range of biological processes and is commercially available. These factors have led to the experimental use of this protein and make it an attractive candidate for further clinical and industrial applications. In this study, we analyze the biological function and compare the thermostability of recombinant SCF^1–165^ and SCF^1–141^ proteins by assessing their ability to enhance the viability of human leukemia cells and mediate downstream biochemical pathways. The C-terminal sequence present in SCF^1–165^ (N-STLSPEKDSRVSVTKKPFMLPPVA-C) but absent in SCF^1–141^ is predicted to function as a flexible loop. However, the possible role of this protein domain in the biological function or thermostability of SCF^1–165^ has not been well characterized.

In a previous study, Wen et al. [20], explored the activity of a glucanase with a 10 kDa deletion from the C-terminus and found that this truncated protein possessed more industrially-favorable properties than the full-length protein, including higher specific activity (4–5-fold increase) and greater thermotolerance. Strikingly, the truncated enzyme retained 80% of its activity even after being boiled for 10 minutes. In the present study, we explore whether the absence of the C-terminal sequence of SCF^1–165^ confers similar properties on SCF^1–141^. We use computer modeling to simulate the structures of these 2 forms of the SCF protein and to calculate their thermal stability, allowing further comparison of their thermal stabilities [21]–[23]. Limited proteolytic digestion was also performed to explore the structural thermostability of these 2 forms of the SCF protein [24]–[25].

## Materials and Methods

### Construction of recombinant human SCF expression vectors

The linear DNA fragments encoding rhSCF^1–165^ and C-terminus truncated rhSCF^1–141^ were obtained from pCR4-TOPO (Invitrogen, Grand Island, NY USA) by PCR performed with a sense primer (5′- ATC GATG GAA GGG ATC TGC AGG AAT CGT -3′) and anti-sense primers (3′SCF^1-165^ [5′- TCA GGC TGC AAC AGG GGG TAA CAT AAA 3′] or 3′SCF^1–141^ [5′-TCA AGA AAC CAC ACA ATC ACT AGT TTC 3′] in which the *Cla* I and *Xba* I sites were introduced. The resulting rhSCF^1–165^ or rhSCF^1–141^ cDNA fragment was double digested with *Cla* I and *Xba* I (TaKaRa, Japan), purified by agarose gel electrophoresis, and cloned into pPICZαC to yield pPICZαC/hSCF^1–165^ or pPICZαC/hSCF^1–141^. The procedures for small scale preparation of plasmid, digestion with restriction enzymes, ligation, and transformation all followed the standard methods. PCR was carried out using 2.5 U of *Taq* DNA polymerase (TaKaRa) in a final volume of 50 µl using the following conditions: 95°C for 10 min, 30 cycles (95°C for 60 s, 55°C for 30 s, and 72°C for 60 s) and a final extension at 72°C for 7 min.

### Electroporation of X33 and screening for recombinant strains

The plasmids pPICZαC/hSCF^1–165^ and pPICZαC/hSCF^1–141^ were linearized with *Sac* I and transformed into yeast *Pichia pastoris* strain X33 using the electroporation method according to the supplier's instruction. Transformed cells were then plated onto YPDS containing 100 µg/mL zeocin and incubated at 30°C for at least 3 days. Single colonies were transferred simultaneously onto Minimal Methanol Medium (MM) and Minimal Dextrose Medium (MD) plates to test their methanol utilization phenotype. The MM and MD plates were incubated at 30°C for 2 days to distinguish between the Mut^s^ and Mut^+^ recombinants. Recombinant strains containing the SCF^1–141^ or SCF^1–165^ gene were screened by colony PCR. Several clones with the Mut^+^ phenotype that expressed the maximal levels of hSCF^1–165^ or hSCF^1–141^ were chosen in small-scale expression and stored in YPD containing 15% glycerol for further scale-up cultures.

### Overexpression of rhSCF^1–141^ or rhSCF^1–165^ in yeast

Selected colonies of zeocin-resistant transformants were inoculated into 5 mL of Yeast Extract-Pepttone-Dextrose (YPD) broth (1% yeast extract, 2% peptone, 2% glucose) containing 100 µg/mL zeocin at 30°C, grown to stationary phase, and used to inoculate 300 mL of Buffered Glycerol-complex Medium (BMGY). After incubation at 30°C with shaking at 100 rpm, the cells were centrifuged at 3000 x *g* for 10 min and the pellets resuspended in 1000 mL of Buffered Methanol-Complex Medium (BMMY) to OD_600_ of 1. The cells were allowed to grow for 72 h at 30°C, and methanol was added every 24 h to a final concentration of 0.5–1% (v/v) to induce expression of the target protein. After 72 h, cells were removed by centrifugation at 3000 x *g* for 5 min, and the supernatants were collected. For recombinant protein detection, the supernatants were analyzed by 15% (w/v) SDS-PAGE. Secretion of the mature protein is expected to result in protein glycosylation. The construct contains the yeast α-factor promoter, which directs the secretion of rhSCF, followed immediately by the sequence for mature rhSCF beginning with the glutamate at amino acid position 1.

### Purification of rhSCF^1–141^ and rhSCF^1–165^ from yeast

Culture supernatants were applied to a phenyl column (GE Healthcare, Piscataway, NJ USA) pre-equilibrated with buffer (0.01 M sodium phosphate, 1.5 M ammonium sulfate; pH 6). After loading, the column was washed with the same buffer and eluted with a linear gradient of 1.5–0 M ammonium sulfate in the same buffer. The active fractions (5 mL) were collected using a flow rate of 60 mL/h. After centrifugation at 20 000 x *g* for 30 min at 4°C, each sample was loaded onto a Q-Sepharose (2.6×20 cm) column pre-equilibrated with 0.01 M sodium phosphate buffer, pH 8 using an AKTA explorer 10S system. Fractions containing rhSCF were eluted with the same buffer containing 1 M NaCl and collected at a flow rate of 120 mL/h. The eluted solution containing rhSCF was concentrated using filter devices with centrifugation at 3000 x *g* for 60 min at 4°C. Concentrated solutions containing rhSCF were loaded onto Sephadex G-75 columns pre-equilibrated with phosphate-buffered saline (PBS) (137 mM NaCl, 2.7 mM KCl, 4.3 mM Na_2_HPO_4_ and 1.47 mM KH_2_PO_4_; pH 7.4). Fractions containing rhSCF were collected and analyzed by 15% SDS–PAGE using Coomassie brilliant blue staining. Western blotting with goat anti-His antibody (1∶1000) (Clontech, Mountain View, CA USA) was used to detect rhSCF with 6-His tag overexpressed from culture supernatant derived from *pichia* transformants carry hSCF^1–141^-6His tag or hSCF^1–165^-6His tag. The amount of purified rhSCF was determined by BCA protein analysis using bovine serum albumin (BSA) as a standard. Aliquots of recombinant proteins were stored in PBS buffer at −20°C and were thawed at 4°C before use in cell viability assays or Western blot analysis for downstream signaling effect.

### Cell lines

The human leukemia cell line TF-1 was purchased from Bioresource Collection and Research Center (Hsinchu, Taiwan) and grown in RPMI-1640 supplemented with 10% fetal bovine serum and 1–5 ng/mL recombinant IL-3. Cells were cultured at 37°C in a humidified chamber containing 5% CO_2_.

### Cell viability assay

Cell viability was measured using the Alamar blue assay (Invitrogen, Grand Island, NY USA) according to the manufacturer's instructions. Briefly, 10 000 TF-1 cells were plated into each well of a 96-well plate, followed by incubation at 37°C in a CO_2_ chamber with media changes every 3 days. Various concentrations of rhSCF were added to the wells. After 5 days, 10 µL of Alamar blue was added to each well incubated and at 37°C for 24 h. The cell viability (% of controls) was calculated using the formula as follows: [rhSCF-treated sample (OD_570_–OD_600_)/untreated sample (PBS) (OD_570_–OD_600_)]×100 Cell suspensions were maintained in 10% FBS as controls (n>6).

### Effects of rhSCF^1–141^ and rhSCF^1–165^ on downstream signaling targets MAPK and Akt

Molecules involved in downstream signaling regulation of rhSCF were examined using Western blot analysis. TF-1 cells were grown at 5×10^5^ cells on 10 cm plates, washed, and starved in 0.5% fetal bovine serum (FBS) medium for 20 to 24 hours, then stimulated with recombinant IL-3 and various concentrations of rhSCF. For Western blot analysis, cells were harvested and lysed with lysis buffer (1% NP-40, 50 mmol/L Tris-HCl [pH 7.4], 0.25% sodium deoxycholate, 150 mmol/L NaCl, 1 mmol/L EGTA, 1 mmol/L PMSF, 1 mg/mL leupeptin, 1 mmol/L Na_3_VO_4_, 1 mmol/L NaF). The unsolubilized material was removed by centrifugation for 10 minutes at 13 000 rpm. Samples were electrophoresed by SDS-PAGE and then transferred onto a PVDF membrane (Immobilon-P; Millipore GmbH, Eschborn, Germany). The membrane was blocked with 5% nonfat dry milk powder in phosphate-buffered saline with 0.1% Tween-20 for 1 hr at room temperature. After blocking, the membrane was incubated with the primary antibody (Antibodies against phospho-MAPK(Thr202/Tyr204), MAPK, phospho-Akt(Ser473), and Akt were all purchased from New England BioLabs (Beverly, MA); β-actin was from Sigma (Sigma-Aldrich, St Louis, MO). The corresponding HRP-conjugated secondary antibody was applied to the membrane for 1 hr to highlight the specific signal. HRP substrate (Millipore, Billerica, MA, USA) was added onto blot membrane to show the pattern, and images were acquired using the LAS-3000 imaging system (Fujifilm, Japan). Blot images were quantified using Multi-Gauge software (Fujifilm). Results are expressed as the ratio of intensity of the protein of interest to that of β-actin from the same sample.

### Circular dichroism (CD) spectroscopy

The thermal stability of the wild-type and truncated forms of rhSCF was determined by measuring temperature-induced changes in ellipticity. CD spectroscopic studies were carried out using an AVIV CD400 spectrometer (Aviv Biomedical, Inc. Lakewood Township, NJ USA) at temperatures of 25–95°C. Samples of purified rhSCF^1–141^ or rhSCF^1–165^ (300 µg/mL) were analyzed in 100 mM sodium phosphate buffer (pH 7.0). Spectra were collected from 190 to 250 nm in 0.5 nm increments, and each spectrum was blank-collected and smoothed using the software package provided with the instrument. Sample cells with 2 mm pathlengths were used for far-UV CD analysis. The spectra were measured 3 times at each temperature. The molar ellipticity θ of each sample was determined using the expression θ = θ_obs_×M_r_/(10×c×I), where θ_obs_ is the observed ellipticity (millidegrees), *M*_r_ is the molecular weight of the protein, *c* is protein concentration (mg/mL), and l is the pathlength (cm).

For use as controls, recombinant SCF^1–141^ and SCF^1–165^ proteins were resuspended in 100 mM sodium phosphate buffer (pH 7.0) at 25°C to yield final concentrations of 300 µg/mL. For experimental groups, recombinant SCF^1–141^ and SCF^1–165^ were treated under the same conditions except heating at 70°C for 10 minutes or 90°C for 15, 30,or 60 minutes. Circular dichroism spectroscopy parameter settings were as follows: detection wavelength range, 190–250 nm; detection pitch, 0.5 nm; and scan speed, 10 nm/min.

### Molecular dynamics simulation and modeling

In this study, we used computer software to simulate, explain, and predict the structural changes that might occur in SCF^1–141^ and SCF^1–165^ after heating. Homology-modeled structures of SCF^1–165^ were predicted using Accelrys Discovery Studio 2.5. In addition, we explained the experimental findings and established a theoretical model through a simulation to explore these structural changes. The structure of SCF was searched in the protein data bank, and the molecule was identified as SCF^1–141^ (PDB ID: 1SCF) through amino acid sequence comparison. However, since the structure of SCF^1–165^ is unknown, SCF^1–141^ was used as a template to simulate the structure of SCF^1–165^, to which amino acids 142–165 were added. We also used a computer software to calculate the structural stability of SCF^1–141^ and SCF^1–165^ at 25°C and 2000 ps.

#### Calculation of the discrete distance between atoms (root mean square value)

Software was used to calculate the discrete distance between atoms before and after heating to compare the thermal stability of SCF^1–141^ and SCF^1–165^ at 25°C, 70°C, and 90°C for 10 ns.

#### Structural collapse figure

Simulations of the structural variability of SCF^1–141^ at 1–10 ns were carried out at 25°C, 70°C, and 90°C.

### Limited proteolytic digestion

Trypsin digestion of rhSCF^1–141^ and rhSCF^1–165^proteins was carried out in 0.1 M-Tris/HCl buffer, pH 8.2, containing 25 mMCaCl_2_. Purified rhSCF^1–165^or rhSCF^1–141^, each 15 µg, was incubated with trypsin at 25°C or 90°C for 10 minutes, or preheated at 90°C 10 minutes, 90 minutes, or 120 minutes and then treated with trypsin for 10 minutes at 25°C or 90°C. After the digestion times indicated, samples were taken and subjected to SDS-PAGE analysis. The digestion products were then separated by SDS-PAGE on a 15% gel and stained with Coomassie blue.

### Statistical analysis

Continuous data are presented as the mean ± SD. The dose-dependent relationship between concentration of rhSCF and cell viability was measured using Spearman correlation coefficient (r_s_). The differences in cell viability between the 2 treatment groups (rhSCF^1–141^ vs. rhSCF^1–165^) and the other factors (temperature and time) were detected using two-way analysis of variance (ANOVA) with interaction. Pairwise post-hoc tests using Bonferroni correction were applied if ANOVA findings were significant. Statistical analyses were performed using SAS software version 9.2 (SAS Institute Inc., Cary, NC). A two-tailed P<0.05 indicated statistical significance.

## Results

### Construction of the rhSCF expression vector

**Figure 1:**
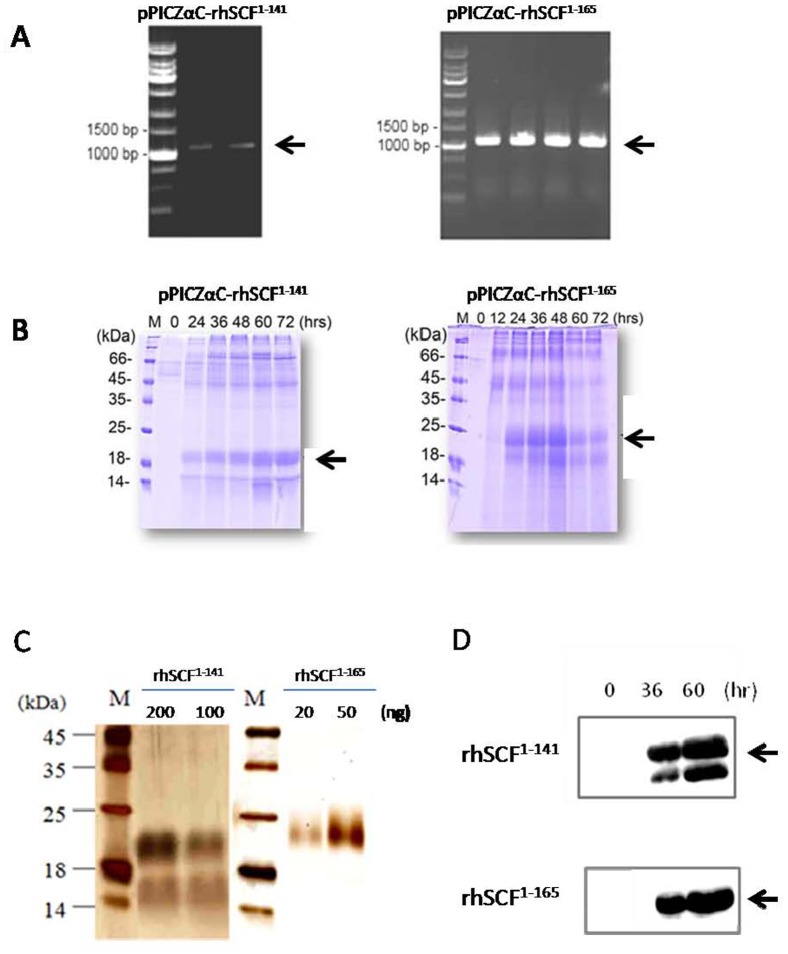
The expression and purification of rhSCF^1–141^ and rhSCF^1–165^. (A) PCR analysis of pPICZαC-rhSCF^1–141^ (1011 bp) and pPICZαC/SCF^1–165^ (1083 bp) using *5′AOX I* and *3′AOX I* primers (arrow). (B) Coomassie blue-stained SDS-PAGE of rhSCF^1–141^ and rhSCF^1–165^overexpressed from *Pichia* culture supernatants. Lane M: molecular weight markers. Numbers above the lanes represent hours after methanol induction. Arrows indicate the overexpressed rhSCF^1–141^ or rhSCF^1–165^ (C) Silver-stained 15% SDS–PAGE of purified rhSCF^1–141^ and rhSCF^ 1–165^ produced in *P. pastoris*. Lane M, molecular weight markers. Lanes 1 and 2, purified rhSCF^1–141^ (200 and 100 ng, respectively); Lanes 3 and 4, purified rhSCF^1–165^ (20 and 50 ng, respectively). (D) Western blot analysis of rhSCF^1–141^-6His and rhSCF^1–165^-6His from culture supernatants. The recombinant proteins were 6xHis tagged and detected with anti-His antibodies.

Two variants of the hSCF gene, a full-length hSCF coding region (aa 1–165) and a truncated hSCF cDNA (aa 1–141), were constructed and inserted into the *P. pastoris* expression vector pPICZαC. In the recombinant plasmid, the hSCF gene was placed downstream of the α-factor promoter and leader sequences in the same ORF. Restriction enzyme analysis and DNA sequencing confirmed that the rhSCF gene was correctly inserted into pPICZαC (Fig. 1A).

### Expression and characterization of rhSCF^1–141^ and rhSCF^1–165^ in *P. pastoris*

The plasmids pPICZαC-rhSCF^1–141^ and pPICZαC-rhSCF^1–165^ were integrated into the alcohol oxidase promoter (*AOX* I) locus of the *P. pastoris* chromosome. Methanol induced the expression of the rhSCF^1–141^ and rhSCF ^1–165^ proteins, appearing on SDS-PAGE as a double band with molecular weights of about 21 and 24 kDa are arrows indicated(Fig 1B).

### Purification of rhSCF^1–141^ and rhSCF^1–165^

Fractions eluted from phenyl column chromatography containing recombinant rhSCF^1–141^ or rhSCF^1–165^ was further purified by Q–Sepharose Fast Flow and Sephadex G-75 column chromatographies. SDS–PAGE of the resulting eluent revealed 2 major bands with molecular weights of approximately 21 and 24 kDa, corresponding to purified rhSCF ^1–141^ and rhSCF ^1–165^, respectively (Figure 1C). The estimated molecular weights of SCF^1–141^ and SCF^1–165^ (based on amino acid sequences) are 15–16 and 18–19 kDa, respectively. Thus, the bands at about 21 kDa (Figure 1 C, lanes 1 and 2 upper band) and 24 kDa (Figure 1C, lanes 3 and 4) are likely the glycosylated forms of rhSCF^1–141^or rhSCF^1–165^. Purified rhSCF^1–141^ still had a minor band around 14 kDa might be the non-glycosylated form (Fig 1C, lanes 1 and 2 lower band). Both are consistent with the result as shown in the western blots using anti-His antibody (Figure 1 D).

### Viability of cells treated with rhSCF^1–141^ and rhSCF^1–165^

**Figure 2:**
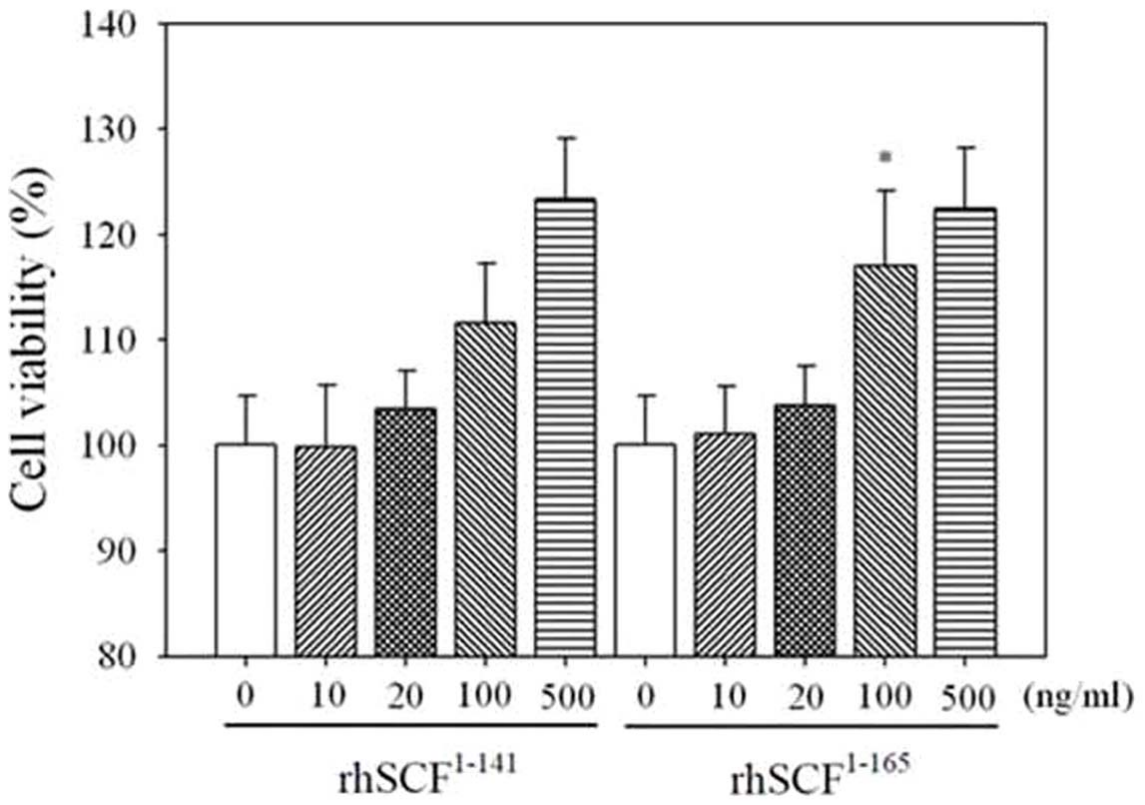
The effect of rhSCF^1–141^ or rhSCF^1–165^ on TF-1 cell viability. Cells were incubated with rhSCF^1–141^ or rhSCF^1–165^ in PBS (pH 7.4) at the concentrations indicated. Results are expressed as mean ± SD (n>6). The viability of untreated control cells was set as 100%. *P<0.05, **P<0.01, ***P<0.001

SCF has been reported to play a role as a survival factor in myeloid cells, preventing cell death via c-Kit [26]. To determine whether SCF treatment affected the viability of TF-1 cells, cell viability assays were performed. rhSCF^1–141^ and rhSCF^1–165^ had a dose-dependent positive effect on human TF-1 cell viability (rhSCF ^1–141^, r_s_ = 0.838, P<0.001; rhSCF ^1–165^, r_s_ = 0.825, P<0.001). TF-1 cells were incubated with 10, 20, 100, or 500 ng/mL rhSCF^1–141^ or rhSCF^1–165^. Cells treated with 100 ng/mL showed significant differences in viability between rhSCF^1–141^ and rhSCF^1–165^ (Figure 2) (P = 0.047).

### Effect of rhSCF^1–141^ and rhSCF^1–165^ on downstream signaling pathways involving MAPK and AKT

**Figure 3:**
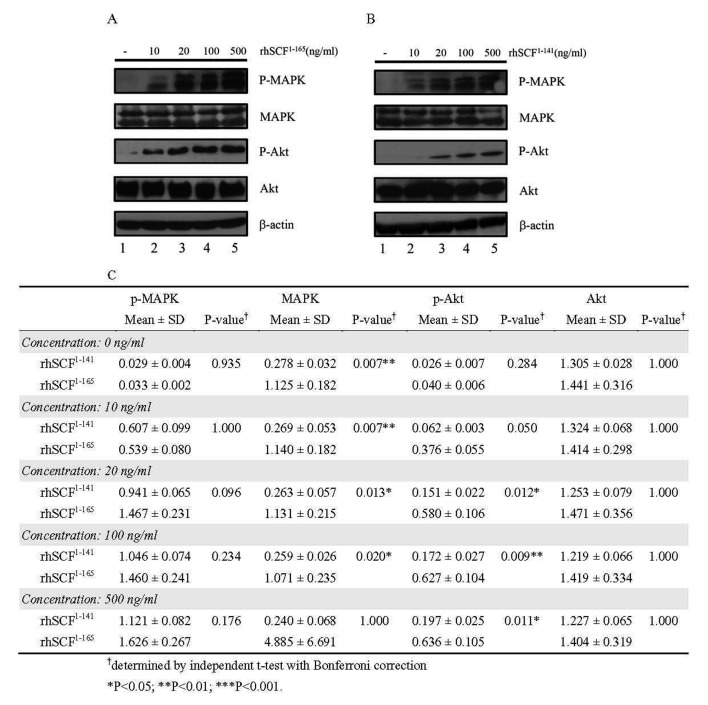
Effects of rhSCF^1–141^ and rhSCF^1–165^ on downstream signaling targets MAPK and Akt. Cells were incubated with rhSCF^1–141^ or rhSCF^1–165^ in PBS (pH 7.4) at the concentrations indicated. A and B are Western blots using the indicated antibodies. Similar results were obtained in 3 independent experiments. C is the quantitation results of the Western blots shown in A and B.

To further establish the biological activity of rhSCF^1–141^ and rhSCF^1–165^, we investigated the expression and phosphorylation of MAPK and AKT in cells treated with rhSCF^1–141^ and rhSCF^1–165^ using Western blotting. Our data show that the levels of pMAPK and pAkt increased in a concentration-dependent manner with either rhSCF^1–141^ or rhSCF^1–165^ treatment (Figure 3A, B). Relative MAPK expression was significantly higher in cells treated with rhSCF^1–165^ than in those treated with rhSCF^1–141^ at concentrations of 0, 10, 20, and100 ng/mL. The relative levels of p-Akt were significantly higher in cells treated with rhSCF^1–165^ than those treated with rhSCF^1–141^ at concentrations of 20, 100, and 500 ng/mL.

### Thermostability of rhSCF^1–165^ and rhSCF^1–141^

**Figure 4:**
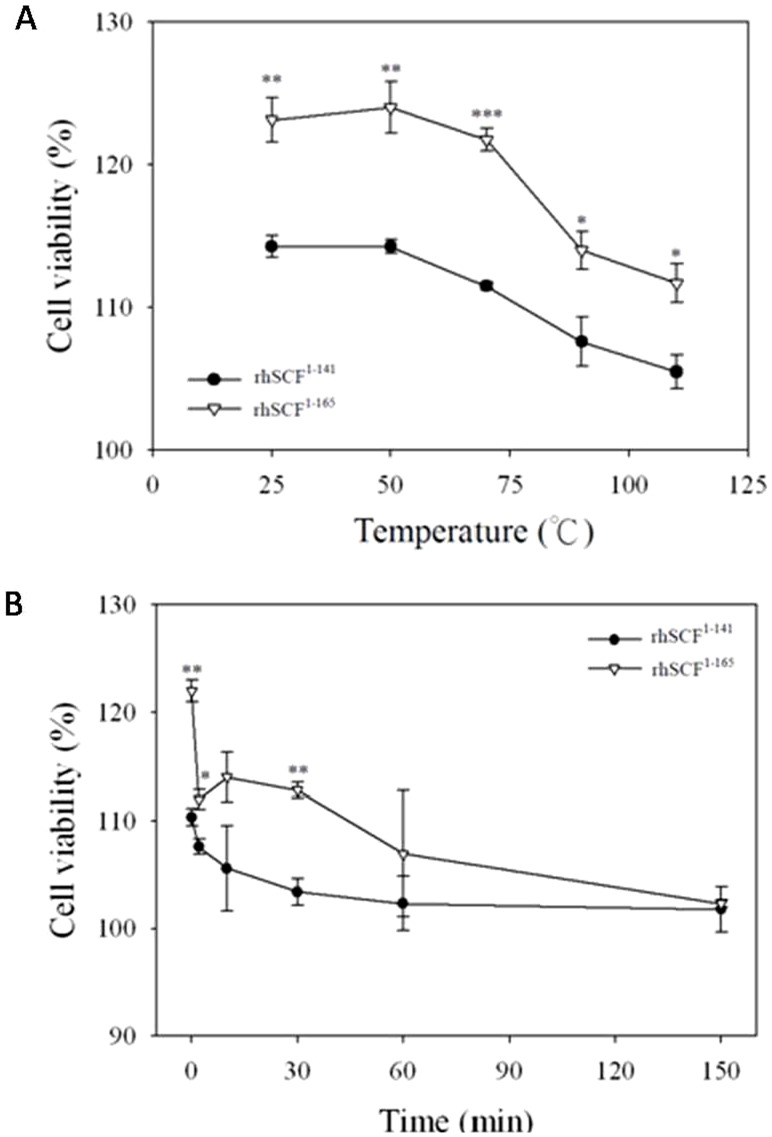
The effect of temperature and time on the viability of TF-1 cells treated with rhSCF^141^ or rhSCF^165^. (A) The effect of temperature on the viability of TF-1 cells treated with rhSCF^1–141^ or rhSCF^1–165^. TF-1 cells were incubated with 100 ng/mL rhSCF^1–141^(solid circle) or rhSCF^1–165^ (hollow triangle) in PBS (pH 7.4) for 10 minutes and the cell viability assayed. (B) Thermostability of rhSCF^1–141^ and rhSCF^1–165^ over time as indicated by cell viability. TF-1 cells were incubated with 100 ng/mL rhSCF^1–141^(solid circle) or rhSCF^1–165^ (hollow triangle) in PBS (pH 7.4) at 90°C for the times indicated. Results are expressed as mean ± SD (n = 3). *P<0.05; **P<0.01; ***P<0.001

We investigated and compared the thermostability of rhSCF^1–141^ and rhSCF^1–165^ at different temperatures. Two-way ANOVA showed a significant difference in viability between cells treated with rhSCF ^1–165^ and rhSCF ^1–141^ (P<0.001) and between those treated at different temperatures (P<0.001). After Bonferroni correction for multiple comparisons, the viability of cells treated with rhSCF ^1–165^ was significantly higher than that of cells treated with rhSCF ^1–141^ at all temperatures tested (Figure 4A). These data suggest that the biological activity of rhSCF^1–141^ is less than that of SCF^1–165^ after heat treatment.

### Thermostability of rhSCF^1–165^ and rhSCF^1–141^ over time

To determine how long the effect of treatment with 100 ng/mL of rhSCF^1–141^ or rhSCF^1–165^ could be maintained at 90°C, we performed cell viability assays over a time course up to 150 minutes (Figure 4B). The results of two-way ANOVA showed both the protein used for treatment and the time of heat treatment significantly affected cell viability (both P<0.001). Our results indicate that the differences in viability between cells treated with rhSCF ^1–165^ and rhSCF ^1–141^ varied with the time of heat treatment (P = 0.007). Using Bonferroni correction for multiple comparisons, we observed that the viability of cells treated up to 30 minutes was significantly higher in those treated with rhSCF ^1–165^ than with rhSCF ^1–141^ (P = 0.002).

### CD spectrometric analysis of rhSCF^1–165^and rhSCF^1–141^

**Figure 5:**
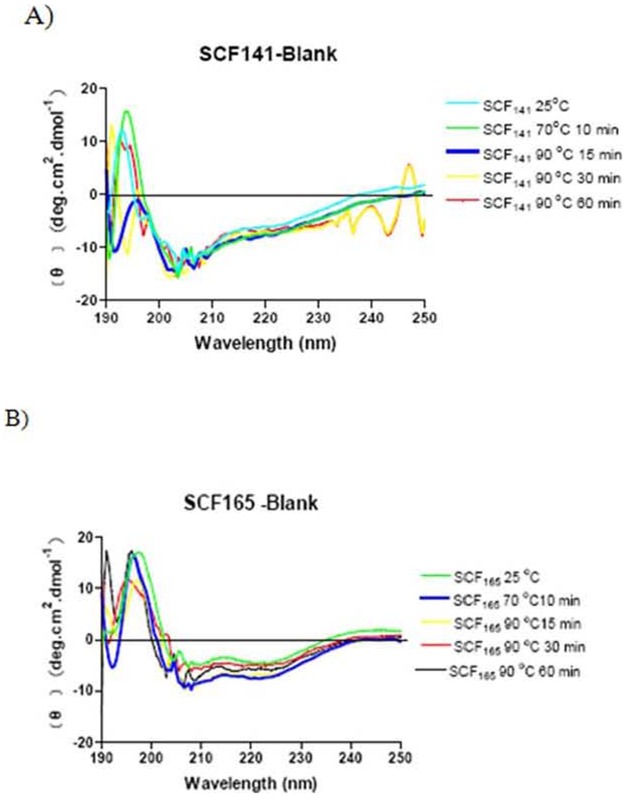
Temperature-dependent changes in circular dichroism spectrum from 190 to 250 nm of (A) rhSCF^1–141^ and (B) rhSCF^1–165^. Conditions: 300 µg/mL in 100 mM phosphate buffer (pH 7.0); 1, 25°C as control; 2, 70°C for 10 min; 3, 90°C for 15 min; 4, 90°C for 30 min; and 5, 90°C for 60 min.

Structural changes in the wild-type (full-length) SCF^1–165^and truncated SCF^1–141^ proteins were analyzed using CD spectroscopy. The far-UV (190–250 nm) CD spectra of full-length and truncated SCF proteins were monitored at temperatures ranging from 25–90°C (Figures 5A and 5B). The CD spectra of rhSCF^1–165^ that was heat-treated at 70°C for 10 min and at 90°C for 15–60 min were identical at 195 nm. The shapes of the bands at 195 nm were similar to those of the unheated control. Conversely, rhSCF^1–141^ treated at 90°C for 15 or 30 minutes had negative bands at 195 nm, suggesting that the β-sheet structures had changed as a result of thermal denaturation.

### Molecular modeling

**Figure 6:**
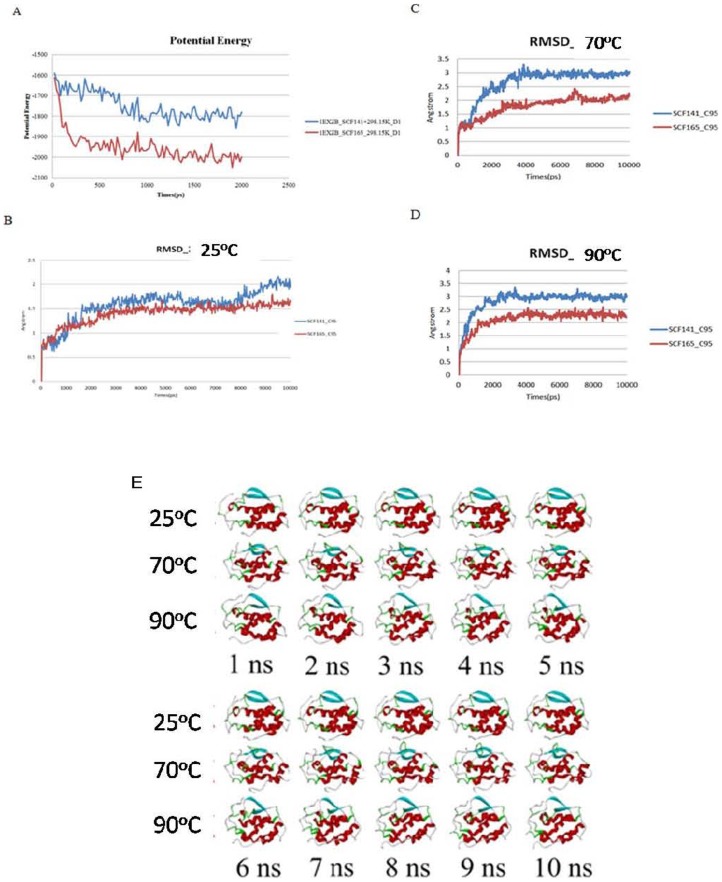
Thermostability study of SCF^1–141^ and SCF^1–165^ by molecular modeling simulation. (A) Potential energy of SCF^1–141^ and SCF^1–165^. The root mean square deviation (RMSD) of SCF^1–141^ and SCF^1–165^ at (B) 25°C; (C) 70°C; and (D) 90°C. (E) Snapshots of the evolving SCF^1–141^ structure at different time points at the indicated temperatures.

A search for the 3D structure of SCF in the protein data bank (PDB) revealed that only the 3D structure of SCF^1–141^has been resolved by the X-ray diffraction method. Therefore, our molecular simulation strategy used SCF^1–141^ as the template. Because the C-terminus of SCF^1–165^ has extra amino acids (142 to 165), simulation software was used to predict its structure. After completing the simulation, we calculated the potential energy (Figure 6A) and the interatomic discrete distance (Root Mean Square Deviation, RMSD) and compared those values between SCF^1–165^ and SCF^1–141^ (Figure 6B, C, and D). We found that after heating 70°C, and 90°C, the discrete interatomic distance of SCF^1–165^ was smaller than that of SCF^1–141^, indicating that recombinant SCF^1–165^ has greater thermal stability than recombinant SCF^1–141^. A snapshot of the temperature-induced structural changes in SCF^1–141^ is presented in Figure 6E.

### Limited proteolytic digestion

**Figure 7:**
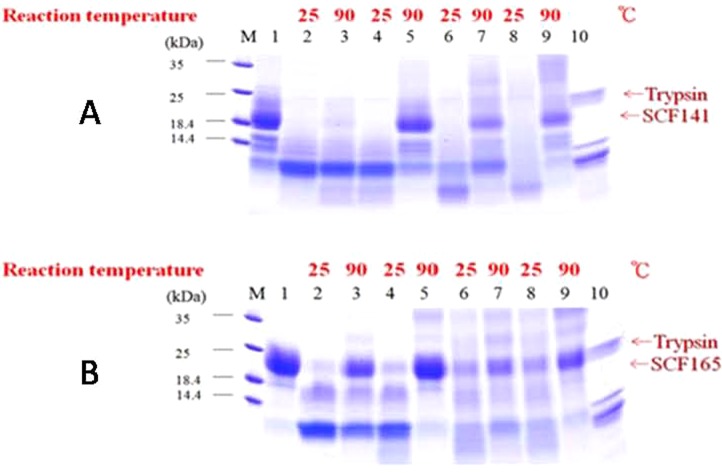
SDS-PAGE analysis of limited proteolytic digestion of rhSCF^1–141^ and rhSCF^1–165^. Purified (A) rhSCF^1–141^ or (B) rhSCF^1–165^ (15 µg each in 0.1 M-Tris/HCl buffer, pH 8.2) was incubated with trypsin at 25°C or 90°C for 10 minutes (lanes 2 and 3), or preheated at 90°C 10 minutes (lanes 4 and 5), 90 minutes (lanes 6 and 7), or 120 minutes (lanes 8 and 9) and then treated with trypsin for 10 minutes at 25°C or 90°C as indicated above. Lanes 1 and 10 are rhSCF and trypsin only.

We used limited proteolytic digestion to investigate the structural stability when digest proteins in their native or denatured configuration. As shown in Figure 7, rhSCF^1–141^ degrades faster than rhSCF^1–165^ and is more susceptible to trypsin digestion, indicating that SCF^1–165^ is more thermostable than SCF^1–141^.

## Discussion

Both rhSCF^1–141^ and rhSCF^1–165^ enhanced TF-1 cell viability in a dose-dependent manner. The biological activity of rhSCF^1–165^ (100 ng/mL) was significantly greater than that of rhSCF^1–141^ after 10 minutes of heat treatment at 25, 50, 70, 90, and 110°C and after heat treatment at 90°C for 0, 2, and 30 minutes. In addition, circular dichroism spectral analysis indicated that β-sheet structures in rhSCF^1–141^ but not rhSCF^1–165^ changed after 15 or 30 minutes of incubation at 90°C. All of these data indicate that SCF^1–165^ is more heat-stable than SCF^1–141^.

The relationship between SCF structure and function has been studied by comparing the activity of a variety of deletion mutants [17], including proteins with missing segments, N- or C-terminal truncations, and disulfide bond alterations. Proliferation and receptor binding activity were found to be deficient in mutants with deletions in or near the disulfide bonds. Forms that included the full length of the extracellular domain were observed to be fully active in both proliferation and receptor binding. Interestingly, *E.coli-*derived SCF^1–141^ was also fully active in both of these functions. Our results are in agreement with those of Langley et al. [17], indicating that *P. pastoris*-derived SCF^1–141^ and SCF^1–165^ both enhance TF-1 cell viability.

We further established that rhSCF is biologically active by analyzing its effects on several downstream targets. Signaling events downstream of the KIT receptor are well documented. In human hematopoietic cells, SCF induces activation and/or recruitment of major kinases such as PI3-kinase, Src kinases (Fyn and Lyn), and JAK2, and various adaptor molecules (Grb2, Grap, Vav, and CRKL) via their SH2 domain. These events result in various molecular associations via SH2, SH3, or PH domains, which in turn trigger the activation of different pathways. Among the signaling cascades that are activated are the Ras/Raf/MEK/MAPK and the PI3K/AKT/RPS6K pathways [27], [28]. Our data show that the levels of pMAPK and pAkt increased in a concentration-dependent manner with either rhSCF^1–141^ or rhSCF^1–165^ treatment, indicating that these recombinant proteins are biologically active (Figure 3). The relative levels of p-MAPK and Akt did not differ between cells treated with rhSCF^1–141^ and rhSCF^1–165^. Relative MAPK expression was significantly higher in cells treated with rhSCF^1–165^ than in those treated with rhSCF^1–141^ at concentrations of 0, 10, 20, and100 ng/mL, and the relative levels of p-Akt were significantly higher in cells treated with rhSCF^1–165^ than those treated with rhSCF^1–141^ at concentrations of 20, 100, and 500 ng/mL. These results indicate that rhSCF^1–165^ treatment affected the expression of MAPK and the phosphorylation of Akt to a greater extent than did rhSCF^1–141^.

We observed that purified or culture supernatant of *P. pastoris-*derived SCF^1–141^ appeared as double bands in silver staining or western blot analysis (Fig. 1C or 1D). The double band might be caused by differential glycosylation or non-glycosylation form of the SCF protein. Such an effect could be confirmed in future studies by deglycosylation enzyme treatment. In a preliminary test, we observed the greatest difference in cell viability (Figure 2) at a concentration of 100 ng/mL of SCF. We then designed a series of thermostability experiments that included a fixed concentration of SCF (100 ng/mL), fixed time (10 minutes), and different incubation temperatures (25, 50, 70, 90, and 110°C). For treatment with either rhSCF^1–141^ or rhSCF^1–165^, incubation at 25–70°C for 10 minutes did not decrease cell viability. However, at temperatures of 90–110°C, cell survival rates gradually declined with increasing temperature. Cell survival rates were higher after treatment with rhSCF^1–165^ than with rhSCF^1–141^, regardless of temperature. For the short heating time of 10 min, rhSCF^1–165^ appears to be more heat-stable than rhSCF^1–141^. At a fixed concentration (100 ng/mL) and temperature (90°C), incubation of cells with either rhSCF^1–141^ or rhSCF^1–165^ for 0–30 min resulted in cell viabilities similar to those observed with non-heated SCF. When the heating time was extended to 30–150 minutes, the cell viabilities decreased to those observed without SCF treatment (control group, 100% survival). Together, these results demonstrate that both full-length soluble SCF^1–165^ and truncated SCF^1–141^ enhance cell viability, but SCF^1–165^ seems to be more stable than SCF1^-141^. We speculate that SCF activity remained after heating because the two intrachain disulfide bonds in the SCF monomers that maintain biological activity were not disrupted by heating, suggesting that beside S-S bond, the C-terminus also contributes to the thermal stability of SCF.

In this study, we also investigated the effects of heat on SCF structure using CD spectroscopy and limited proteolysis. Protein molecules have aromatic amino acids whose cyclic structures absorb UV light (λ = 100 nm–400 nm). The degree of circularly polarized light absorption depends on the wavelength of light and is affected by the secondary structure of the protein. Thus, CD scanning of a protein at different wavelengths of light generates a spectrum that can be used to determine its secondary structure. We observed that the CD spectra of rhSCF^1–165^ that was heat-treated at 70°C for 10 min and at 90°C for 15, 30 and 60 min were identical at 195 nm, the wavelength characteristic of the β-sheet spectrum [29], [30]. The shapes of these bands at 195 nm were also similar to that of the unheated control. Conversely, rhSCF^1–141^ treated at 90°C for 15 or 30 minutes had negative bands at 195 nm, suggesting that the β-sheet structures had changed as a result of thermal denaturation. These results indicate that the secondary structure of SCF^1–165^ was more thermally stable than that of SCF^1–141^. Similarly, heat treatment of rhSCF^1–141^ affected its susceptibility to proteolytic digestion to a greater extent than that of rhSCF^1–165^, indicating that SCF^1–141^ is more heat labile than SCF^1–165^ (Figure 7). Furthermore, the change in the alpha and beta structures of SCF^1–141^ owing to its heat treatment can be observed in snapshots of the evolving SCF^1–141^structure at different time points at different temperatures.

We used molecular modeling software to predict the RMSD values of the protein based on theoretical chemistry. Again, we found that SCF^1–165^ had a higher thermal stability than SCF^1–141^. Based on these results, we speculate that recombinant SCF^1–165^ had a higher thermal stability than SCF^1–141^ because the additional 14 amino acids in the C-terminus of SCF^1–165^ play an important role in the structural stability of the protein. This hypothesis deserves further exploration.

Stem cell factor is a glycoprotein with 3 potential sites of N-linked glycosylation: Asn^65^, Asn^72^, and Asn^120^. We are interested in characterizing the glycosylation state of *P. pastoris*-derived SCF to determine whether glycosylation is important for the biological function of SCF. In future studies, we will express rhSCF in *E.coli* and in *P. pastoris* and compare the glycosylation state and activity between SCF derived from these 2 species.
